# Methamphetamine use and HIV risk behavior among men who inject drugs: causal inference using coarsened exact matching

**DOI:** 10.1186/s12954-020-00411-1

**Published:** 2020-09-21

**Authors:** Mehdi Noroozi, Peter Higgs, Alireza Noroozi, Bahram Armoon, Bentolhoda Mousavi, Rosa Alikhani, Mohammad Rafi Bazrafshan, Ali Nazeri Astaneh, Azadeh Bayani, Ladan Fattah Moghaddam

**Affiliations:** 1 Social Determinants of Health Research Center, University of Social Welfare and Rehabilitation Sciences, Tehran, Iran; 2 Department of Public Health, School of Psychology & Public Health, La Trobe University, Bundoora, Victoria Australia; 3 Department of Neuroscience and Addiction, School of Advanced Technologies in Medicine, Tehran University of Medical Sciences, Tehran, Iran; 4 Social Determinants of Health Research Center, Saveh University of Medical Sciences, Saveh, Iran; 5 Psychosis Research Center, Department of Psychiatry, University of Social Welfare and Rehabilitation Sciences, Tehran, Iran; 6 Department of Nursing, School of Nursing, Larestan University of Medical Sciences, Larestan, Iran; 7 Department of Psychiatry, University of Social Welfare and Rehabilitation Science, Tehran, Iran; 8 Student Research Committee, School of Allied Medical Sciences, Shahid Beheshti University of Medical Sciences, Tehran, Iran; 9 Department of Nursing, Faculty of Nursing and Midwifery, Tehran Medical Sciences, Islamic Azad University, Tehran, Iran

**Keywords:** Methamphetamine, HIV, Risk Behavior, Coarsened Exact Matching, MWID

## Abstract

**Background:**

Understanding the association between methamphetamine (MA) use and HIV risk behavior among people who inject drugs (PWID) will assist policy-makers and program managers to sharpen the focus of HIV prevention interventions. This study examines the relationship between MA use and HIV risk behavior among men who inject drugs (MWID) in Tehran, Iran, using coarsened exact matching (CEM).

**Methods:**

Data for these analyses were derived from a cross-sectional study conducted between June and July 2016. We assessed three outcomes of interest—all treated as binary variables, including distributive and receptive needle and syringe (NS) sharing and condomless sex during the month before interview. Our primary exposure of interest was whether study participants reported any MA use in the month prior to the interview. Firstly, we report the descriptive statistics for the pooled samples and matched sub-samples using CEM. The pooled and matched estimates of the associations and their 95% CI were estimated using a logistic regression model.

**Results:**

Overall, 500 MWID aged between 18 and 63 years (mean = 28.44, SD = 7.22) were recruited. Imbalances in the measured demographic characteristics and risk behaviors between MA users and non-users were attenuated using matching. In the matched samples, the regression models showed participants who reported MA use were 1.82 times more likely to report condomless sex (OR = 1.82 95% CI 1.51, 4.10; *P* = 0.031), and 1.35 times more likely to report distributive NS sharing in the past 30 days, as compared to MA non-users (OR = 1.35 95% CI 1.15–1.81). Finally, there was a statistically significant relationship between MA use and receptive NS sharing in the past month. People who use MA in the last month had higher odds of receptive NS sharing when compared to MA non-users (OR = 4.2 95% CI 2.7, 7.5; *P* = 0.013).

**Conclusions:**

Our results show a significant relationship between MA use and HIV risk behavior among MWID in Tehran, Iran. MA use was related with increased NS sharing, which is associated with higher risk for HIV exposure and transmission.

## Background

There has been a growing trend of methamphetamine (MA) use in Iran over the last decade [1]. Bio-behavioral surveys conducted among PWID have shown increases in self-reported past month MA use from 42% in 2011 to 61% in 2013 [2]. Needle and syringe sharing and condomless sex place PWID at increased risk for HIV and other infections [3–6] with international studies showing this risk is increased further when MA is the primary drug used [7, 8]. There is also evidence to show PWID who use MA are more likely to share needles when injecting, and to not consistently use sterile injection equipment when compared to PWID who do not report MA use [9]. Furthermore, MA is a stimulant that enhances sex drive, lowers inhibitions and increases self-confidence. Some people who use opioids or receive opioid maintenance treatment (OMTs) report MA use as a way to increase sex drive and performance [10]. As a result, people who use MA may be more likely to engage in HIV risk behaviors, such as having condomless sex and exchanging sex for money [11]. There is evidence showing MA injecting can negatively impact the effectiveness of harm reduction programs including needle and syringe programs (NSPs), and opiate maintenance therapy (OMT) [12].

Previous international studies have assessed the relationship between MA use, unsafe sexual and injection risk behaviors among PWID using observational methods [7]; however, observational studies have methodological limitations (i.e., confounders) [13]. Logistical and ethical issues mean that experimental studies using MA are difficult to conduct therefore the use of novel approaches exploring the impact of MA use on risk taking behaviors are required. Little is also known about the effect of MA use on injection and sexual behavior among PWID in developing countries, especially Iran [14]. To address these limitations in the literature, we performed Coarsened Exact Matching (CEM) to analyze data collected from interviews with PWID.

In the present study, we applied CEM to construct statistically equivalent groups of survey respondents who were susceptible and not susceptible to MA use. Understanding this association will assist policy-makers and program managers to sharpen the focus of their HIV prevention interventions. Therefore, the main of this study was to measure the effect of MA use on the injecting risk behaviors of MWID in Tehran, Iran.

## Methods

### Study sample and procedures

Data for these analyses were collected using a cross-sectional survey with MWID conducted between June and July 2016 in Tehran, Iran. The study design and setting have been described in detail elsewhere [15] with PWID being recruited using convenience and snowball sampling from drop-in centers located in southern Tehran. PWID were eligible for inclusion in the study if they met the following criteria: (1) were aged over 18 years, (2) reported injecting drugs at least once in the month before interview, and (3) could provide informed consent to participate in the study. Participants completed a modified version of the behavioral surveillance surveys (BSS) used previously in Iran [16].

The questionnaire included modules on socio-demographic characteristics (age, marital status, education level, and living status), drug use history, injecting risk behaviors including receptive syringe sharing (obtaining and using a syringe after being used by someone else), and distributive syringe sharing (giving someone else a syringe after it has already been used) and high risk sexual behaviors (condomless anal and/or vaginal intercourse). Data collection and written informed consent was obtained by trained research staff.

We assessed three outcomes of interest, all treated as binary variables, including distributive and receptive needle sharing and condomless sex with any type of sexual partner in the month before interview. The receptive sharing variable was derived from a survey question which asked participants: “in the last 30 days, with how many people did you use a needle after they injected with it?” The responses were dichotomized into any receptive sharing in the last month (yes vs. no). The condomless sex variable was derived from survey questions which asked participants about engaging in sex with a partner without using a condom in the last month (including regular, casual or commercial sex partners). Our primary exposure of interest was whether study participants had reported MA use in the month prior to the interview. Based on exposure status, we created two groups: exposure (i.e., MA use) and control (i.e., no reported MA-use) groups. Verbal and written consent procedures were provided to all participants before the survey was administered.

### Analysis methods

Firstly, we report the descriptive statistics for the pooled sample and matched sub-samples using CEM. CEM is a nonparametric method of preprocessing data to control for some or all of the potentially confounding influences of pretreatment control variables by reducing imbalance between the treated and control groups. We used CEM to match the groups based on certain covariates and thus made statistically equivalent comparison groups to estimate the independent effect of MA use on both injection and sexual risk-taking behaviors. The CEM created comparable subgroups based on covariates including housing status, income, current OMT, education level, and whether participants access NSP. These behaviors were assessed because they were found to be associated with injection risk-taking and risky sexual behaviors [17–19], and therefore, may be confounders of the association between MA use and specific HIV risk behaviors. Using CEM, we allocated every study participant into one of a specified set of stratum in which all were exactly matched on a set of coarsened variables. Matched members were then assigned a weight specific to that stratum and representative of the proportion of all members present in that stratum. Then, we calculated a statistical measure called L1 distance. We calculated the overall imbalance using the L1 statistic before and after matching (indicating minimal imbalance between the two comparison groups). Then, the pooled and matched estimates of the association and their 95% CI were estimated using a logistic regression model. All data analysis was performed using Stata V.12

## Results

The sample included 500 male PWID, aged between 18 and 63 years (mean = 28.44, SD = 7.22). The majority of participants (73%) were currently single, had not completed high school (72%), and 64% reported their initiation into injecting drug use before 25 years of age. Additionally, 27.3% were homeless, and 67.3% had monthly incomes of less than $150. Over three quarters (78%) of participants reported receiving OMTs for at least 2 months and 70% reported accessing a needle syringe program (NSP) in the past 6 months.

As seen in the matched sample “receptive” and “distributive” syringe sharing in the month before interview was reported by 44% and 24%, of PWID respectively. In addition, 73% of participants reported “inconsistent condom use with any sex partner” in the previous 30 days. Also, 70% of participants reported MA use in the month before interview. The results of the subgroup analysis are presented in Table 1.

**Table 1:** Demographic characteristics and risk behaviors (matched and unmatched samples) among PWID, Tehran, 2016

Variables	Pooled sample *N* = 500 (100%)	Matched sample *N* = 370 (100%)
	Number	
Age		
< 30 ≥ 30	275 (55) 225 (45)	220 (59) 150 (41)
Completed years of education		
< 5 years 5–8 > 8	160 (32) 200 (40) 140 (28)	110 (29) 150 (40) 110 (31)
Marital status		
Married Single	180 (36) 320 (64)	100 (27) 270 (73)
Age of first drug injecting (years)		
< 25 ≥ 25	290 (58) 210 (42)	240 (64) 130 (35)
Income (Toomans)		
< 500,000 500,000–1,000,000 > 1,000,000	190 (38) 180 (36) 130 (26)	120 (32) 130 (35) 120 (28)
Housing status		
Homeless Stable home	150 (30) 350 (70)	100 (27) 270 (73)
Receiving opioid maintenance treatments (OMT)	380 (76)	290 (78)
MA use	360 (72)	260 (70)
Condomless sex with any partner in past month	300 (60)	270 (73)
Receptive syringe sharing in past month	220 (44)	160 (43)
Distributive syringe sharing in past month	120 (24)	90 (24)

Table 2 presents estimates of the effect of MA use on both injecting and sexual risk taking in the matched and unmatched samples. In the matched samples, the regression models showed participants who reported MA use were 1.82 times more likely to have condomless sex (OR = 1.82 95% CI 1.51, 4.10; *P* = 0.031) and 1.35 times more likely to report distributive needle sharing in the past 30 days, when compared to MA non-users (OR = 1.35 95% CI 1.15–1.81; *P* = 0.012). Finally, there was a statistically significant relationship between MA use and reported receptive syringe sharing in past month. People who used MA last month had higher odds of receptive syringe sharing when compared to those not reporting any MA use (OR = 1.36 95% CI 1.11, 1.85; *P* = 0.013).

**Table 2:** Estimates of effect of MA use on injecting and sexually risk behaviors in matched and unmatched samples by logistic regression model among PWID, Tehran, 2016–2017

Outcome	Unmatched sample OR (CI 95%)	Matched sample OR (CI 95%)
Condomless sex		
MA users MA non-users	2.85; 95% CI 1.57–4.17 1	1.82; 95% CI 1.51–4.10 1
Receptive syringe sharing in past month		
MA users MA non-users	1.66 (1.41–1.91) 1	1.36 (1.11–1.85) 1
Distributive syringe sharing in past month		
MA users MA non-users	1.51 (1.34–1.86) 1	1.35 (1.15–1.81) 1

## Discussion

The results of this study suggest that in this group of PWID, MA use increases the risk of past month condomless sex and receptive and distributive syringe sharing. Collectively, these findings are consistent with previous studies that have identified significant associations between condomless sex and MA use [20, 21]. PWID reporting recent MA use were more likely to have condomless sex than MA non-users. An Australian study found that most people (72%), who use MA frequently, reported being sexually active but only 35% of them reported regular condom use with their casual partners in the last month [22]. A study of men referred to one of three HIV prevention and testing program in California (*N* = 1839) found high rates (11.1%) of amphetamines and sildenafil citrate (Viagra®) use [23]. HIV infection and other sexually transmitted diseases were higher among those who used both amphetamines and Viagra® compared to those who used only one or neither drug. Viagra® use was associated with insertive anal intercourse, and methamphetamine was associated with receptive anal intercourse [23]. An earlier study conducted among men who have sex with men in the same city found taking Viagra® at the same time as MA was associated with insertive anal intercourse [24].

Our findings show that MA increases receptive syringe sharing in the past month. This finding is consistent with other studies [3, 25]. Our study revealed an independent relationship between syringe sharing and MA use and suggests that PWID are at increased risk for exposure to HIV and other blood-borne infections. Our results regarding drug-related HIV-risk behaviors suggest that risk practices related to injecting may reflect another important aspect of HIV transmission among people who use MA.

The production of crystal MA in clandestine laboratories is highly profitable and easy to hide, with evidence suggesting that law enforcement interventions have had a limited effect on the production or importation to main international markets in North America and Europe [26]. Since 1996, precursor chemicals for MA production have been scheduled and highly regulated in the US resulting in temporary perceived disturbances in MA markets, with little effect on MA use and related health problems among MA users [27, 28]. Thus, it is essential to implement evidence-based HIV prevention, drug treatment, and harm reduction interventions to address MA use among PWID and reduce high risk behaviors in this population [29, 30]. Our study shows that there is an independent relationship between syringe sharing and MA injection and suggests that individuals who inject MA are more susceptible to HIV and other blood-borne infections infection.

Consistent with previous studies [3, 31], we found that participants who used MA were more likely than MA non-users to report distributive needle sharing in the month before interview.

The results of our study provide several insights for future studies investigating injection-related risk behavior especially among younger (under 25 years) MA injectors. Considering that MA users are more likely to experience barriers to accessing harm reduction and HIV prevention services, interventions and approaches that improve secondary syringe distribution (i.e., receiving supplies from peers who access NSPs) are essential [32]. Studies indicate that models of syringe distribution, including fixed and outreach-based services performed by or catered specifically to youth, are advantageous in other settings [33]. The development of youth-friendly supervised injecting facilities acceptable to those injecting MA are also worthy of further investigation as they decrease syringe sharing among hard-to-reach and hidden populations [34].

In addition, interventions that improve positive peer norms about harm reduction efforts among MA-using injecting networks may be efficient at decreasing risk-taking behavior and supporting the use of HIV prevention and other health services [35]. Creating effective HIV prevention approaches by working with MA injectors must consider the injecting practices and health problems experienced by these individuals [36].

Our results show that those who injected MA report inadequate access to sterile syringes. Further research investigating the most prevalent individual, social, and structural obstacles experienced by those who inject MA is required. However, the present study indicates that NSP offered by adult injectors can positively influence HIV prevention interventions for this population [37, 38].

There is some suggestion that MA injectors do not feel comfortable when using the HIV prevention interventions that are provided for users of other drugs [12]. Therefore, MA injectors themselves must be prioritized in the development of new interventions, including placing services in areas frequented by young people who use MA and adopting peer-based staffing models for future NSP expansion.

Although there are structural barriers which are associated to the ability of individuals to access HIV prevention services, other individual factors and social influences exist that prevent MA injectors from accessing sterile syringes [39]. Previous studies have shown that MA injectors are more susceptible to inject within friendship groups, and this may increase the risk of sharing of syringes and other injecting equipment [40]. It is recommended that future studies investigate the barriers to service utilization and to determine how individual, social, and structural barriers increase HIV-related harms.

Our study had several limitations including the cross-sectional design and the use of self-report data. Since our data are not a random sample from the population under investigation, generalizing the results to other drug-users or other settings is limited.

Previous studies have indicated that PWID self-reports are valid [41, 42], but, since syringe sharing behavior is stigmatized, it may be underreported [43].

HIV prevention, harm reduction, and education programs are required to reduce HIV risk taking by people who use MA. Given there are currently few evidenced informed interventions for MA use among PWIDs in Iran our results provide essential information for expanding risk reduction programs and interventions.

## Conclusion

Our results show a significant relationship between MA use and HIV risk behavior among PWID in Tehran, Iran. MA use was related with an increased risk of syringe sharing, which is associated with greater risk for HIV infection transmission. Current law enforcement measures have failed to control the MA market and therefore the development of specific drug treatment and harm reduction interventions addressing specific needs of PWID who use MA are urgently needed. It is recommended that novel, PWID-driven interventions, including the development of current services to resolve the requirements of this population be developed to decrease HIV transmission among people who inject methamphetamine.

## Data Availability

The datasets used and/or analyzed during the current study are available from the corresponding author on reasonable request.

## Abbreviations

BSS: Behavioral surveillance surveys
CEM: Coarsened exact matching
DICs: Drop-in centers
MA: Methamphetamine
MWID: Men who inject drugs
NSPs: Needle and syringe programs
OMT: Opioid maintenance treatment
OR: Odds ratios
PWID: People who inject drugs
